# miRNA-Dependent CD4^+^ T Cell Differentiation in the Pathogenesis of Multiple Sclerosis

**DOI:** 10.1155/2021/8825588

**Published:** 2021-01-08

**Authors:** Justyna Basak, Ireneusz Majsterek

**Affiliations:** Department of Clinical Chemistry and Biochemistry, Medical University of Lodz, Lodz, Poland

## Abstract

Multiple sclerosis (MS) is characterized by multifocal lesions, chronic inflammatory condition, and degenerative processes within the central nervous system (CNS) leading to demyelination. The most important cells involved in its pathogenesis are those which are CD4^+^, particularly proinflammatory Th1/Th17 and regulatory Treg. Signal cascades associated with CD4^+^ differentiation are regulated by microRNAs (miRNAs): short, single-stranded RNAs, responsible for negative regulation of gene expression at the posttranscriptional level. Several miRNAs have been consistently reported as showing dysregulated expression in MS, and their expression patterns may be elevated or decreased, depending on the function of specific miRNA in the immune system. Studies in MS patients indicate that, among others, miR-141, miR-200a, miR-155, miR-223, and miR-326 are upregulated, while miR-15b, miR-20b, miR-26a, and miR-30a are downregulated. Dysregulation of these miRNAs may contribute to the imbalance between pro- and anti-inflammatory processes, since their targets are associated with the regulation of Th1/Th17 and Treg cell differentiation. Highly expressed miRNAs can in turn suppress translation of key Th1/Th17 differentiation inhibitors. miRNA dysregulation may result from the impact of various factors at each stage of their biogenesis. Immature miRNA undergoes multistage transcriptional and posttranscriptional modifications; therefore, any protein involved in the processing of miRNAs can potentially lead to disturbances in their expression. Epigenetic modifications that have a direct impact on miRNA gene transcription may also play an important role.

## 1. Introduction

Neurologic autoimmune disorders, such as multiple sclerosis (MS), are characterized by multifocal lesions, chronic inflammatory condition, and degenerative processes within the central nervous system (CNS). Although a wide range of symptoms is observed, they are most commonly associated with the glia hyperplasia, axon damage, and degeneration of myelin sheath. These dysfunctions lead to significant cognitive impairment and disability, especially among young adults [1–3]. Demyelination is a major syndrome of MS; however, its background is still not fully understood. Long-standing research on MS indicates that the demyelination process is largely associated with immunopathology. Some of the factors known to be associated with the development of MS include macrophage infiltration of the brain and spinal cord, autoreactive antibodies, complement activation, and increased production of proinflammatory cytokines [4, 5]. However, autoreactive CD4^+^ cells migrating to CNS lesions are also believed to be key players in the pathogenesis of MS [6].

It is currently postulated that immune system disorders may be caused by epigenetic mechanisms such as small, noncoding RNAs, especially alterations in microRNA (miRNA) expression [7]. MicroRNAs are short, single-stranded RNAs that are responsible for negative gene expression regulation at the posttranscriptional level. Due to their properties, miRNAs can play a role in many cellular processes, such as maintenance of homeostasis, differentiation of cells, and tissue development, and their activity can be determined by numerous physiological or pathological factors [8]. It has been found that miRNAs are involved in inducing inflammation by targeting lymphocyte differentiation and secretion of proinflammatory cytokines [9]. miRNA dysregulation can occur as a result of many processes, including disorders in the biogenesis pathway and transcriptional regulation as well as epigenetic modifications.

This paper discusses the significance of CD4^+^ cell differentiation in regulating inflammatory and autoimmune processes, as well as the key miRNAs involved in the pathogenesis of MS. In addition, the final part examines the potential causes of miRNA dysregulation.

## 2. The Role of CD4^+^ Cells

**Figure 1:**
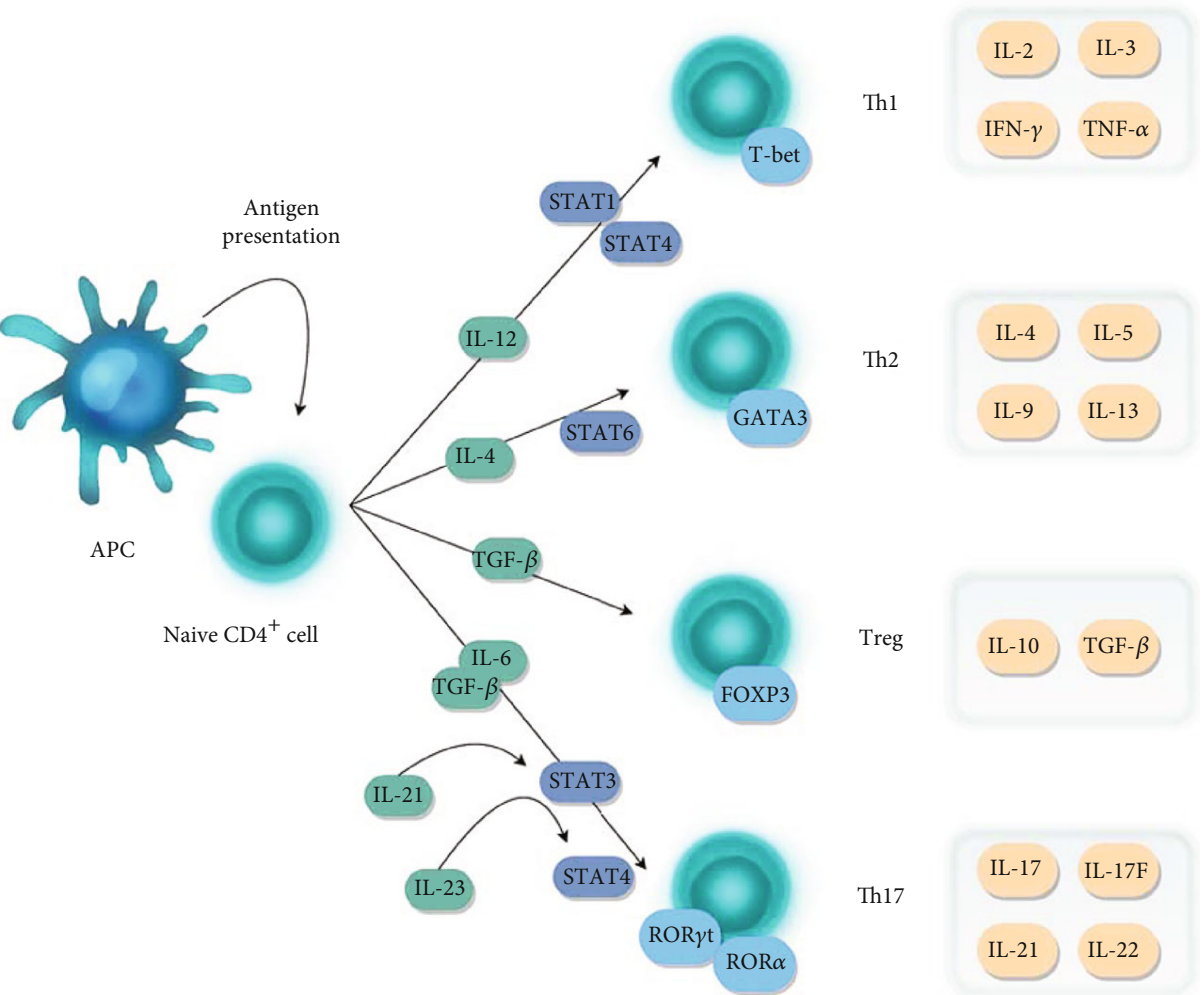
Naive CD4^+^ cells are activated via antigen-presenting cell (APC) and differentiate into several subtypes, *viz.* Th1, Th2, Treg, or Th17, under the influence of specific cytokines. Differentiation of Th1 cells is stimulated by the presence of IL-12, which activates the signaling pathways associated with STAT proteins (STAT1 and STAT4) and T-bet transcription factor. Mature Th1 cells secrete proinflammatory cytokines: IL-2, IL-3, IFN-*γ*, and TNF-*α*. Th2 cells differentiate under the influence of IL-4 and STAT6, which activate GATA3 transcription factor and stimulate the production of cytokines, including IL-4, IL-5, IL-9, and IL-13. Treg cell differentiation is dependent on the presence of TGF-*β*, and the typical transcription factor expressed by Treg is FOXP3. TGF-*β* and IL-10 are also effector cytokines secreted by Treg cells. The presence of TGF-*β* and IL-6 stimulates the differentiation of Th17 cells through STAT3 signaling pathways, as well as transcription factors ROR*γ*t and ROR*α*. In addition, Th17 differentiation can be induced by alternative pathways stimulated with IL-21 and IL-23 (activating STAT4). Major effector cytokines include IL-17, IL-17F, IL-21, and IL-22.

The most common form of MS, relapsing-remitting MS (RRMS), is characterized by alternating exacerbations of symptoms and remission periods. Relapse events are largely associated with acute inflammatory response and accumulation of immune cells in the white matter and myelin tracks of grey matter in the CNS. The most important players in the development of MS are antimyelin CD4^+^ T cells, CD8^+^ T cells, and B cells. However, among these factors, CD4^+^ cells appear to play a major role in the pathogenesis of MS, being an essential source of interleukins [10, 11]. Activation of naive CD4^+^ cells via antigen stimulation leads to differentiation into several subtypes including Th1, Th2, Th17, or Tregs. Each CD4^+^ cell subtype indicates a different cytokine pattern and triggers distinct effects (Figure 1) [12].

Th1 cells, due to their proinflammatory properties, were initially recognized as the main factor involved in the development of MS [11]. The proinflammatory nature of Th1 cells results from their role in immune responses against intracellular pathogens such as viruses and bacteria [12]. Although tumor necrosis factor *α* (TNF-*α*), IL-2, and IL-3 are all effector cytokines associated with the Th1-dependent response, the most significant Th1 cytokine is interferon *γ* (IFN-*γ*) [13]. The primary role of IFN-*γ* is to stimulate macrophage activity in the fight against the pathogen, but according to research, it may also play a role in autoimmunity, since elevated IFN-*γ* levels have been observed in patients with MS and other autoimmune disorders [14, 15]. Production of IFN-*γ* and development of Th1 are stimulated by IL-12 and activation of STAT4, a member of the signal transducer and activator of transcription (STAT) protein family [16, 17]. Another STAT family member, STAT1, is also involved in Th1 cell differentiation by mediating the activation of T-bet, belonging to the T-box protein family. T-bet is particularly important for the differentiation of Th1 cells because it has been shown to play a role in reprogramming the maturation of naive T cells from Th2 to Th1 subtype, thereby influencing the nature of the immune response [18, 19]. Th2 cells are involved in the fight against extracellular pathogens and participate in a humoral type of immune response through activation of B cells [13]. The Th2 cytokine profile is associated with, i.e., IL-4, IL-5, IL-9, and IL-13. IL-4 is a pivotal effector cytokine, as well as a major factor responsible for inducing Th2 cell differentiation by activating the STAT6-associated signal pathway and GATA3 transcription factor. In addition, IL-4 mediates the inhibition of Th1 cell proliferation, which determines the anti-inflammatory properties of Th2 cells. Mutual inhibition of Th1 and Th2 cells leads to the formation of a specific immune balance, which is particularly important for the proper functioning of the immune system [20].

Recently, however, most studies have focused on Th17, which is considered a pivotal agent of autoimmunity. Th17 cells, like other types of lymphocytes, are involved in the response against pathogens; however, their role may be associated with the generation of significantly greater inflammation than in the case of other cells. Th17 cells also indicate greater proliferative potential than Th1; therefore, they are considered a more pathogenic factor than other types of lymphocytes [21, 22]. Interestingly, it has been shown that Th17 antagonizes Th2 cells but also Th1 cells, because the cytokines responsible for promoting Th17 cell proliferation, especially transforming growth factor *β* (TGF-*β*), inhibit the differentiation of other types of lymphocytes, by acting on IFN-*γ* and T-bet, among others [23]. Of all the Th17 cytokines, IL-17 is responsible for most of the proinflammatory effects: it is found to be a crucial factor in the development of experimental autoimmune encephalomyelitis (EAE), an animal model of MS [24]. In addition, Th17 cells secrete several other cytokines that are responsible for inducing inflammation, including IL-17, IL-17F, IL-21, and IL-22 [25]. Studies have also shown that the activity of IL-17 and IL-22 contributes to the migration of Th17 cells through the blood-brain barrier (BBB) to the acute myelin sheath lesions in patients with MS [26, 27].

The key pathway responsible for Th17 cell differentiation is regulated by TGF-*β* and IL-6 [28, 29]. However, it has been shown that maintaining and stabilizing the proinflammatory features of Th17 cells require additional signals from different cytokines, such as IL-23 and IL-21, which modulate alternative signaling pathways [30, 31]. TGF-*β*, along with IL-6 or IL-21, is responsible for activating STAT3, while IL-23 is involved in the STAT4-induced signal cascade [32]. STAT family proteins associated with Th17 cell differentiation are responsible for the activation of two retinoid nuclear receptors, ROR*γ*t and ROR*α*, that directly bind to the IL-17 gene promoter and trigger its transcription [33, 34]. Both isoforms are crucial for regulating Th17, because ROR*γ*t overexpression and coexpression with ROR*α* have been shown to increase Th17 proliferation significantly, while suppressing ROR*γ*t and ROR*α* expression completely removes EAE symptoms by inhibiting Th17 differentiation [34].

Interestingly, the interleukins associated with Th17 cells also have a negative effect on the development of regulatory T cells (Tregs) secreting TGF-*β* and IL-10. The primary role of Tregs is associated with suppressing excessive inflammatory responses and inhibiting autoreactive cells. Although Tregs express a range of transcription factors, FOXP3 seems to be crucial for maintaining their anti-inflammatory functions and regulating the balance between Th17 and Treg differentiation. It appears that expression of FOXP3 is induced in the presence of TGF-*β* but inhibited by IL-6. Studies have shown that IL-6 significantly contributed to regulating the balance between Tregs and Th17 and promoting the proliferation of the latter; however, production of Th17 is possible even after IL-6 depletion, confirming that other cytokines as mentioned above are also involved in this process [31, 35].

The factors involved in controlling CD4^+^ cell differentiation are tightly regulated to allow proliferation of the appropriate cell subtype. As such, miRNAs, being important factors that regulate gene expression, are the subject of many studies.

## 3. miRNA Dysregulated in MS and EAE

Since the first report of the discovery of small RNA molecules, regulating the expression of *lin-4* in *C. elegans*, numerous miRNAs have been identified in humans, plants, animals, and viruses [36]: over 40 000 precursors of miRNAs from 207 organisms have been discovered [37]. The high diversity and abundance of miRNAs make these molecules extremely important cellular regulators, despite the relatively low level of repression. It is estimated that miRNA inhibits the expression level of its targets by less than 50%; however, it should be noted that a single gene could be modulated by several miRNAs and a single miRNA can regulate the expression of hundreds of genes. Research has shown that miRNAs are responsible for regulating approximately 30% of human genes. Interestingly, single miRNAs may target several signaling pathways that ultimately affect the same factor, thus repeatedly amplifying the effect of miRNA regulation: any disturbances in their expression can have significant results [38, 39].

Several miRNAs have been consistently reported as showing dysregulated expression in MS, due to their involvement in the development or control of existing inflammation [40]. Basically, miRNA expression patterns may be elevated or decreased depending on the function of specific miRNAs in the immune system; however, in many cases, the level of expression of a particular miRNA may vary between different tissues or cell types and may even change significantly during remission or relapse. Studies in MS patients and EAE animals indicate that let-7e [41], miR-17 [42], miR-141, miR-200a [43], miR-145 [44], miR-155 [45], miR-223 [46], and miR-326 are upregulated [47], while miR-15b [48], miR-20b [49], miR-26a [50], and miR-30a are downregulated [51] (Table 1). A deficiency in these miRNAs may contribute to the imbalance between pro- and anti-inflammatory processes, since their targets are associated with the regulation of Th1/Th17 and Treg cell differentiation [50]. Highly expressed miRNAs can in turn suppress translation of key Th1/Th17 differentiation inhibitors, such as ETS-1, which is targeted by several miRNAs, including miR-326 and miR-155 [45, 47], as well as some forkhead family proteins, such as FOXO1 or FOXO3, targeted by miR-183C and miR-141/-200a [43, 52]. Furthermore, novel research also indicates that FOXO3 expression may be negatively regulated by miR-155, miR-223, and miR-29b [53]. In contrast, miR-301a participates in promoting Th-17 differentiation by inhibiting the expression of PIAS3, a STAT3 inhibitor, and thus regulating the IL-6/STAT3 signaling pathway [53]. In addition, some upregulated miRNAs, e.g., miR-142, may target proteins that regulate cytokine production. One of the key targets of miR-142 is SOCS1, which inhibits signaling cascades associated with STAT and stabilizes Treg cell proliferation. Its deficiency caused by high expression of miR-142 may therefore contribute to Th1/Th17 and Treg cell imbalance. Furthermore, this miRNA can also target TGF-*β* and adenylate cyclase type 9 (ADCY9), which significantly limits Treg proliferation [54, 55]. Treg cells may also be regulated by miRNA targeting FOXP3, such as miR-24, miR-145, and miR-210. Expression of these miRNAs is usually reduced in Treg cells; nevertheless, overexpression may occur in autoimmune processes [44, 56].

However, among all analyzed miRNAs, miR-155 appears to play a crucial role in the pathogenesis of MS and EAE, as well as in other inflammation- and neurodegeneration-related disorders [57, 58]. It has been proven that miR-155 expression is significantly increased in active lesions in MS patients as well as in various types of immune cells and brain-resident cells, and the level of expression of this miRNA corresponds to high levels of proinflammatory cytokines, suggesting its participation in induction or maintaining inflammation [59]. Furthermore, the role of miR-155 in regulating the immune system is complex, and it can both mediate normal immune responses and trigger chronic inflammation. It has been shown that miR-155 may be involved in the maintenance of Treg cell homeostasis and the susceptibility of other T cells to Treg regulation [59, 60]. Also, the regulation of Th17 proliferation by mir-155 may be more complicated than previously thought, especially in the pathogenesis of MS. Mycko et al. have shown that miR-155-3p can target heat shock proteins such as Dnaja2 and Dnajb1 in EAE mice, whose high expression inhibits the proliferation of myelin-reactive Th17 cells [61]. Moreover, mice with lowered miR-155 levels were less subject to severe EAE symptoms and recovered significantly more quickly than miR-155-sufficient mice [61, 62]. Comparable results in animal studies with EAE were also obtained for several other miRNAs, such as miR-21, miR-223, and miR-326 [46, 47, 63]. miR-223 deficiency probably contributes to reduced penetration of autoreactive Th1/T17 cells into the spinal cord and also reduces the activation of dendritic cells (DC) producing Th17-polarizing cytokines [46, 64]. However, the effect of miR-21 knockout is probably associated with an increase in the activity of Smad7, known to be a negative regulator of the TGF-*β* signal pathway [63]. However, several miRNAs show an opposite trend in the development of EAE, since their overexpression is associated with a milder course of the disease. These miRNAs are associated with reduced expression patterns in MS, and they have been found to target proteins that are directly or indirectly involved in signaling pathways affecting Th/T17 cell differentiation [48, 65]. For instance, miR-15b may silence the expression of O-linked N-acetylglucosamine transferase (OGT), which mediates the regulation of the NF-*κ*B pathway essential for T cell activation via TCR (T cell receptor) [48].

Another frequently reported player in Th17 differentiation is miR-146a, whose deficiency has been shown to increase T cell reactivity and IL-17 secretion during autoimmune response. mir-146a is responsible for silencing the expression of *TRAF6* (TNF receptor-associated factor 6) and *IRAK1*, two factors associated with the NF-*κ*B signaling pathway. Furthermore, miR-146a can also promote anti-inflammatory cytokines, such as IL-4, and suppress transcription of *STAT1*, thereby promoting a Th2-dependent response [66]. Möhnle et al. also have shown that in human T cells, miR-146a regulates the expression of the protein kinase C epsilon (PRKC*ε*), which is responsible for the phosphorylation and activation of STAT4 in the differentiation of Th1 lymphocytes, suggesting that miR-146a may be also involved in inhibiting the Th1 cell-dependent pathway [67]. Moreover, miR-146a plays a significant role in the regulatory properties of Tregs. Treg cells have been shown to inhibit the proliferation of other CD4^+^ T cell subtypes by arresting them in the G1 phase, which is probably mediated by miR-146a. The expression of miR-146a is significantly increased in T cells inhibited by Tregs relative to those that were not; i.e., they did not receive a signal to stop dividing. It is likely that the upregulation of miR-146a in these cells mediates the silencing of IL-2 expression, the most important growth factor for CD4^+^ cells [68]. Interestingly, although numerous studies indicate that miR-146a is an important factor inhibiting the autoimmune response, a significant increase in its expression is observed in active lesions in animals with EAE, which may be the result of its involvement in silencing inflammation [69].

## 4. Disorders of miRNA Expression

miRNA dysregulation may result from the impact of various factors at each stage of their biogenesis. Immature miRNA undergoes multistage transcriptional and posttranscriptional modifications; therefore, changes in any of the proteins involved in the processing of miRNAs can potentially lead to disturbances in their expression. Likewise, an important role may also be played by epigenetic modifications with a direct impact on miRNA gene transcription.

### 4.1. Epigenetic Modifications

The regulation of miRNA expression is largely dependent on the location of the pri-miRNA sequence. Briefly, pri-miRNA sequences may be located in regions between genes (intergenic miRNA) or within genes (intragenic miRNA). While intragenic miRNAs can be found in both introns and exons, intronic miRNAs make up the majority of all miRNAs [70]. The position of the miRNA gene is significant for the process of transcription, as well as the presence of epigenetic modifications. The promoters of intronic miRNAs can be located in genomic regions remote from the gene sequence itself, e.g., in exons. In addition, some intronic miRNAs have promoters independent of the host gene [71]. Transcription of intergenic miRNAs may also be controlled by mechanisms independent of the transcription of protein-coding genes [72]. The independent promoters can impact the expression of miRNA, depending on the tissue and the current condition of the cell [73, 74].

Studies indicate that methylation of promoter or even distant enhancer sequences can have a great impact on the level of miRNA transcription. Therefore, changing the degree of methylation of CpG islands or enhancers can significantly change the miRNA expression profile, especially since a significant proportion of miRNA genes are located within or near CpG islands than in protein-coding genes; the former position is associated with a higher frequency of pre-miRNA methylation [75]. Research indicates that abnormal methylation patterns in close proximity to the miRNA promoter may indeed be associated with a reduction in the expression of mature miRNAs [76].

**Figure 2:**
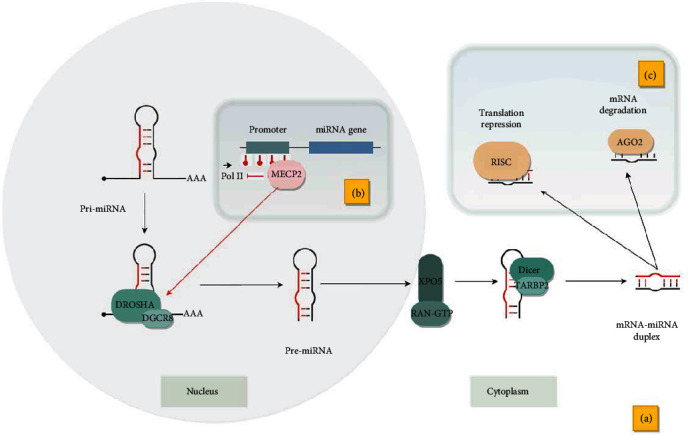
(a) Primary miRNA transcripts (pri-miRNAs) are processed in the nucleus via DROSHA and DGCR8 into pre-miRNAs. Pre-miRNAs are transported by XPO5 and RAN-GTP to the cytoplasm, where they are further transformed by Dicer and TARBP2 into mature molecules. (b) Methylation of the miRNA promoter sequence may have a direct effect on miRNA biogenesis. The MECP2 protein, by binding to methylated sequences, inhibits transcription and allows pri-miRNA processing, which increases the level of mature miRNAs. (c) Mature miRNAs can silence expression of target genes through two main mechanisms: repression of translation through the RISC complex and direct transcript degradation by AGO2.

However, recent studies have indicated an opposite trend. Weber et al. report that MECP2 (methyl-CpG binding protein 2) can have a significant impact on the final result of miRNA methylation: the level of methylation can have a direct impact on miRNA biogenesis (Figure 2). The expression of highly methylated miRNAs is significantly more affected by a change in methylation pattern than low-methylated miRNAs. In addition, by binding to the methylated sequences in miRNA genes, MECP2 interferes with the chain elongation process during transcription; this enables DROSHA and DGCR8 to process pre-miRNA, resulting in an increase in the production of mature miRNA molecules. These findings indicate that methylation of miRNA genes is relevant and may modulate their expression in a variety of ways [77].

miRNA expression can also be modulated by posttranslational modifications of histones (PTM) that can trigger or suppress transcription by interacting with promoter sequences. The PTMs associated with miRNA promoters have been mainly studied in cancers, which has resulted in the discovery of many new dysregulations affecting miRNA expression [78]. Particularly significant modifications include the methylation and acetylation of histone H3 lysine, especially tri-methylation of lysine 4, 9 and 27 (H3K4me3, H3K9me3 and H3K27me3), di-methylation of lysine 9 (H3K9me2) and the acetylation of lysine 9 and 14 (H3K9ac and H3K14ac). While most of these modifications are responsible for inhibiting expression, trimethylation, similarly to acetylation, increases transcription of miRNA [79]. Histone modifications are regulated by the activity of enzymes that add or remove specific groups, directly affecting the gene expression profile. Studies show that histone deacetylase (HDAC) can very quickly modulate miRNA expression and therefore may be an excellent therapeutic target for disorders associated with altered miRNA expression patterns [80]. Although these studies have been based on different types of cancers, it is possible that similar mechanisms may be responsible for the regulation of miRNA in autoimmune diseases. In addition, several miRNAs closely associated with autoimmune diseases have been shown to be regulated by the presence of epigenetic modifications [81, 82]. Acetylation of histones H3 and H4 as well as methylation of CpG islands near the miR-146a gene has been confirmed in B cells and Burkitt's lymphoma cell line (BL), indicating that these modifications may have a significant influence on the regulatory properties of miRNA in the immune system [81].

### 4.2. MicroRNA Processing Pathway

The microRNA synthesis pathway has been quite widely described in the scientific literature. Many proteins involved in this process have been identified, and it therefore seems obvious that disturbances in their expression will affect quantitative changes in miRNAs. Dicer and DROSHA are crucial enzymes involved in the biogenesis of miRNAs. DROSHA processes the primary transcripts (pri-miRNAs) to pre-miRNAs, which are transported to the cytoplasm by exportin 5 (XPO5) and processed by Dicer and TARBP2 to mature miRNA molecules. Numerous studies indicate that Dicer is crucial for the proper functioning of the organism, but due to its other functions, it is not clear whether the disorders related to Dicer deficiency are associated with impaired miRNA activity. Drosha is probably more likely associated with miRNA expression disorders than Dicer, because it is not involved in the processing of other small noncoding RNAs. However, research shows that both DROSHA and Dicer have a significant contribution to miRNA expression, and thus impairment of their function may have a significant effect on immune system activity. It has also been demonstrated that deletion of the Dicer and DROSHA genes leads to dysfunction of lymphocytes [83, 84]. In addition, studies indicate that the levels of Dicer, DROSHA, and DGCR8 proteins are repeatedly increased in patients with MS relative to the control group [85].

miRNA activity can also be dysregulated during the final stages of their processing, i.e., during the assembly of RNA-induced silencing complex (RISC) consisting of Argonaute (AGO) proteins, Dicer, and TARBP2 among others. The proteins in the RISC complex are directly responsible for silencing expression, which can be done through several different mechanisms, such as cleavage of the mRNA target, repression of translation, or mRNA decapping and deadenylation [86]. The mature miRNA has a sequence complementary to the target mRNA, and in animals, the primary mechanism for silencing involves repression of translation by partially matched miRNA. In animals, miRNA targeting is generally based on a short seed sequence with high complementarity, localized in the positions of 2–8 nucleotides at the mRNA 3′-untranslated region (3′-UTR) end, but in some cases, other positions at the 3′-UTR in mRNA sequence may be recognized [87, 88].

The exact mechanism of translation repression has not been explained; however, it is assumed that repression may occur at the initiation stage, as miRNAs have been shown to inhibit the recruitment process of IF6 (the antiassociation factor binding 60S subunit), which prevents the assembly of the 80S ribosome subunit [89]. The AGO protein of the RISC complex can also compete with eIF4E (eukaryotic translation initiation factor 4E) for 5′ cap binding; therefore, it is postulated that miRNAs can silence expression by suppressing initiation of translation or blocking the recycling of ribosomal subunits [90]. Moreover, although translation repression is thought to play a significant role in silencing, there is growing evidence that transcripts subjected to endonucleolytic cleavage can be degraded via the typical mRNA degradation pathway including deadenylation and decapping by specific exonucleases. The human AGO2 protein probably also demonstrates endonuclease activity and may play a role in the process of mRNA cleavage [91]. In addition, AGO2 can recruit further proteins performing deadenylation of poly(A) tails, which is the first stage of degradation of the target mRNA [92].

Due to the complexity of gene silencing mechanisms involving miRNA and the multitude of additional factors involved in this process, any change in the functioning of individual elements may translate into disorders in miRNA activity. Liu et al. found miRNA expression in EAE mice to be significantly affected by Ago2, indicating that the protein is significantly involved in miRNA processing and maintaining its homeostasis, as well as is critical to normal immune function [93]. Earlier studies also have shown that changes in the level of expression of components of the RISC complex are important for inhibiting or increasing miRNA processing. Studies in mice lacking the *Ago2* gene have also found the protein to be necessary for the proper development of the embryo, in particular the neural tube [94, 95]. In addition, it is suggested that RISC complex proteins, mainly AGO2, perform key functions in the reprogramming of naive T lymphocytes into effector cells, since their expression is reduced in maturing lymphocytes. Furthermore, naive T cells with reduced AGO2 expression are much more likely to differentiate into T cells that secrete proinflammatory cytokines [96]. Due to the proven role of these processes in T cell differentiation, it is possible that the accumulation of aberrations in miRNA processing may lead to disturbances in their expression in the development of diseases such as MS.

## 5. Conclusions

The functioning of immune response must be strictly regulated, since every disturbance in such a complicated and delicate system can lead to the progression of illness. Therefore, it is not surprising that miRNAs can significantly modulate the immune response, considering that they are such important components in gene expression regulation. This fact has been confirmed by numerous studies indicating that certain miRNAs influence the differentiation of T cells in the course of MS. As the regulation of miRNA is tissue and cell dependent, further research is needed to fully understand the relationship between miRNAs and observed phenotypes. Moreover, such an approach implies many therapeutic options and also enables the development of better diagnostic methods. The epigenetic modification of miRNA genes is an interesting therapeutic target, due to the wide availability of pharmacological agents such as commonly used enzyme inhibitors involved in DNA methylation or histone modifications. Therefore, it is particularly important to thoroughly understand the basis of miRNA dysregulation and the mechanisms of miRNA gene silencing, as the inhibition of even single miRNAs may show significant pleiotropic effects.

## Figures and Tables

**Table 1:** miRNAs involved in CD4+ T cell differentiation of multiple sclerosis (MS) patients and/or experimental autoimmune encephalomyelitis (EAE) animals.

miRNA	Change in expression	Target	Function	Reference
let-7e	Upregulated in CD4^+^ cells, in EAE animals	IL-10	Promotion of Th1/Th17 differentiation	[41]
miR-15b	Downregulated in CD4^+^ cells, in MS patients and EAE animals	OGT	Inhibition of Th17 differentiation	[48]
miR-20b	Downregulated in blood cells of MS patient and in EAE animals	ROR*γ*t, STAT3	Inhibition of Th17 proliferation	[49]
miR-21	Upregulated in Th17 cells	SMAD7	Promotion of Th17 differentiation	[63]
miR-26a	Downregulated in peripheral blood lymphocytes of MS patients	IL-6	Regulation of Th17/Treg balance	[50]
miR-30a	Downregulated during Th17 differentiation in MS patient and EAE animals	IL-21R	Inhibition of Th17 proliferation	[65]
miR-141 miR-200a	Upregulated in MS patients	FOXO3, GATA3, SMAD2	Regulating Th17 and Treg differentiation	[43]
miR-142	Upregulated in MS patients and EAE animals	TGF-*β* SOCS1 ADCY9	Promotion of Th17 differentiation and inhibition of Treg function	[54, 55]
miR-145	Downregulated in Treg cell, upregulated in blood cells of MS patients	FOXP3 SMAD3	Inhibition of Treg proliferation	[44, 56]
miR-146a	Upregulated in lesions in EAE animals	TRAF6 IRAK1 PRKC*ε*	Inhibition of Th1 proliferation, supporting regulatory mechanisms	[66, 67, 69]
miR-155	Upregulated in MS patients	ETS1 DNAJA2 DNAJB1	Promotion of Th17 differentiation	[59, 62]
miR-183C	Upregulated in pathogenic Th17 cells	FOXO1	Stimulation the production of pathogenic cytokines	[52]
miR-301a	Upregulated in CNS-infiltrating T cells in EAE animals	PIAS3	Promoting Th17 differentiation	[53]
miR-326	Upregulated in EAE animals	ETS1	Promoting Th17 differentiation	[47]

## Data Availability

The data supporting this systematic review are from previously reported studies and datasets, which have been cited. The processed data are available the corresponding author upon request.

## Abbreviations

3′-UTR: 3′-untranslated region
ADCY9: Adenylate cyclase type 9
AGO: Argonaute
APC: Antigen-presenting cell
BBB: Blood-brain barrier
BL: Burkitt's lymphoma cell line
CNS: Central nervous system
EAE: Experimental autoimmune encephalomyelitis
eIF4E: Eukaryotic translation initiation factor 4E
FOXP3: Forkhead box P3
IF6: Antiassociation factor binding 60S subunit
IFN-*γ*: Interferon *γ*
IL: Interleukin
IRAK1: Interleukin 1 receptor-associated kinase 1
MECP2: Methyl-CpG binding protein 2
miRNA: microRNA
MS: Multiple sclerosis.
NF-*κ*B: Nuclear factor kappa-light-chain-enhancer of activated B cells
OGT: O-linked N-acetylglucosamine transferase
PTM: Posttranslational modifications of histones
RISC: RNA-induced silencing complex
ROR: RAR-related orphan receptor
RRMS: Relapsing-remitting multiple sclerosis
SOCS1: Suppressor of cytokine signaling 1
T-bet: T-box transcription factor 21
TCR: T cell receptor
TGF-*β*: Transforming growth factor *β*
TNF-*α*: Tumor necrosis factor *α*
TRAF6: TNF receptor associated factor 6
Treg: Regulatory T cell
STAT: Signal transducer and activator of transcription
XPO5: Exportin 5.
